# Cell lines authentication and *mycoplasma* detection as minimun quality control of cell lines in biobanking

**DOI:** 10.1007/s10561-017-9617-6

**Published:** 2017-03-02

**Authors:** C. Corral-Vázquez, R. Aguilar-Quesada, P. Catalina, G. Lucena-Aguilar, G. Ligero, B. Miranda, J. A. Carrillo-Ávila

**Affiliations:** Andalusian Public Health System Biobank, Avenida Del Conocimiento S/N, 18016 Granada, Spain

**Keywords:** STRs, Cell line authentication, Mycoplasma, Quality control, Biobanking, PCR

## Abstract

Establishment of continuous cell lines from human normal and tumor tissues is an extended and useful methodology for molecular characterization of cancer pathophysiology and drug development in research laboratories. The exchange of these cell lines between different labs is a common practice that can compromise assays reliability due to contamination with microorganism such as mycoplasma or cells from different flasks that compromise experiment reproducibility and reliability. Great proportions of cell lines are contaminated with mycoplasma and/or are replaced by cells derived for a different origin during processing or distribution process. The scientific community has underestimated this problem and thousand of research experiment has been done with cell lines that are incorrectly identified and wrong scientific conclusions have been published. Regular contamination and authentication tests are necessary in order to avoid negative consequences of widespread misidentified and contaminated cell lines. Cell banks generate, store and distribute cell lines for research, being mandatory a consistent and continuous quality program. Methods implementation for guaranteeing both, the absence of mycoplasma and authentication in the supplied cell lines, has been performed in the Andalusian Health System Biobank. Specifically, precise results were obtained using real time PCR detection for mycoplasma and 10 STRs identification by capillary electrophoresis for cell line authentication. Advantages and disadvantages of these protocols are discussed.

## Introduction

The use of cultured cells that acquired the ability to proliferate indefinitely is an extended tool in research laboratories. Cell lines are used as in vitro models of health and disease by retaining many of the properties of the parental tissue or cell type, including disease-specific changes (Christine Alston-Roberts et al. 2010; Shannon et al. 2016). Because of those reasons, cell lines based screening platforms are excellent models to test new therapeutic approaches.

Nowadays is frequent the exchange of established cells between different laboratories as result of groups interactions. That practice involves a high risk of cell lines contamination by two common sources: a microorganism, usually *mycoplasma*, or a foreign cell line. Many cell lines currently used are contaminated with *mycoplasma* and/or are replaced by cells derived for a different origin without researcher knowledge (Capes-Davis et al. 2010; Drexler et al. 2017). The consequences of widespread misidentified and contaminated cell lines are immeasurable and it cannot be ignored by the scientific community (Huang et al. 2017).

*Mycoplasma* contamination of cell cultures was first described in the 1950s (Macpherson 1966). Mycoplasmas and the related Acholeplasmas (both referred as “mollicutes”) are the smallest self replicating bacteria and the most prevalent microbial contaminant of cell. These microorganisms pass through standard 0.22 µm filter, are not affected by commonly used antibiotics in cell mediums and can grow until extremely high titres without producing any turbidity in the supernatants. Between 18 and 31% of cell cultures are contaminated with *mycoplasma* (Macpherson 1966) affecting seriously to the experimental results of cell viability, gene expression, cell morphology and metabolism and growing rate (Nubling et al. 2015). *Mycoplasma* contamination may affect both the scientific results of cell culture-based research and the quality of biological medicines manufactured by cell culture in the biopharmaceutical industry for therapeutic use (Armstrong et al. 2010; Laborde et al. 2010; Volokhov et al. 2011). The common sources of *mycoplasma* contamination are: cross-contamination of cell lines from other *mycoplasma*-positive cell cultures, researchers, laboratory equipment, contaminated reagents, the N_2_ liquid of cryostorage vessels, feeder cell cultures and laboratory animals. Because of the magnitude of this problem a periodic *mycoplasma* detection test must be performed in every cell line manipulated in the laboratory. In fact, scientific journals are requiring free *mycoplasma* cell lines before accepting manuscripts for publication (Geraghty et al. 2014).

Cell line misidentification is the other one of the most serious and persistent problems detected in culture laboratories (Geraghty et al. 2014; Drexler et al. 2017; Huang et al. 2017). Cross-contamination between cell lines may be due to several reasons such as an accidental contact, contaminated mediums or reagents, the use of mitotically inactivated feeder layers or conditioned medium which may carry contaminating and not properly eliminated cells (van Pelt et al. 2003). Besides, a cell line can be replaced by another because of mislabeling or confusion during handling (Christine Alston-Roberts et al. 2010). Because of those reasons, established cell lines need to be authenticated by a reference standard method (Ayyoob et al. 2015).

Different methods for cell lines authentication have been described: chromosomal analysis/karyotyping (MacLeod et al. 2007), isoenzyme analysis (Stacey et al. 1997), multilocus DNA fingerprint analysis (Jeffreys et al. 1985; Stacey et al. 1992), short tandem repeat (STR) profiling (Masters et al. 2001; Butler 2006), polymerase chain reaction fragment analysis (Steube et al. 2008) and sequencing of “DNA barcode” regions (Hebert et al. 2003). The selection of a specific method depends on the researcher’s purpose, the expected resolution and the laboratory’s expertise. By other hand, the discovery of DNA hypervariable regions within genomes has made possible to identify each human cell line derived from a single donor. Jeffreys et al. (1985) demonstrated in 1985 that hypervariable regions, which consist of variable number tandem repeat (VNTR) units from minisatellite DNA, are capable of hybridizing to many loci distributed throughout the genome to produce a DNA “fingerprint”. In spite of the intrinsic difficulties of DNA fingerprint, subsequent advances in the technology have given rise to the use of microsatellite regions consisting of core sequences of 1–6 bp, repeated in a different number in each cell line. Because the polymorphism of STRs are hotspots for homologous recombination events, these markers display many variations in the number of the repeating units between loci in unrelated cell lines (Wahls et al. 1990).

Cell banks generate, store and distribute controlled cell lines. Their activity of stocks testing for *mycoplasma* and authenticity minimizes the contamination risks associated with prolonged passaging (Kiehlbauch et al. 1991). So, the implementing of a consistent quality control in biobanking to guarantee *mycoplasma* free and authentication of cell lines is crucial (Cardoso et al. 2010). In order to establish a cell lines quality control workflow different methods were chosen and results were compared.

## Materials and methods

### Human biological samples

Handling of human biological samples was carried out according to national legal framework [Law on Biomedicine Research (July 2007)]. The samples used were collected following informed consent of the donors and immediately anonymized. Local scientific and ethic committees approved the procedures performed in this work.

### Cell lines supernatants

Twenty-four supernatant samples from tumor cell lines generated by the Biobank were used to *mycoplasma* detection. 100 µl supernatants were heated at 95 °C for 10 min and centrifuged at 1000 g for 5 s to discard cellular debris.

### Conventional PCR *mycoplasma* detection

LookOut *mycoplasma* PCR Detection Kit (Cat. no. MP0035, Sigma-Aldrich, MO, USA) for detection of 19 *mycoplasma* species was used following the manufacturer instructions for *mycoplasma* detection in cell cultures. PCRs were performed using an Eppendorf AG thermocycler. Results were visualized using Agilent DNA 1000 Reagents (Cat. no. 5067-1504, Agilent Technologies, CA, USA) in a 2100 Bioanalyzer (Agilent Biotechnologies, CA, USA).

### Real-time PCR *mycoplasma* detection

LookOut *mycoplasma* qPCR Detection Kit (Cat. no. MP0040, Sigma-Aldrich, MO, USA) for detection of 66 *mycoplasma* species was used following manufacturer instructions for *mycoplasma* detection in cell cultures. PCR inhibition was discarded by an internal control from the kit (ROX labeled). PCRs were performed with specific Taqman probes (FAM labeled) using an ABI 7500 real time PCR thermocycler (Applied Biosystems, Singapore, Asia).

### DNA isolation for STRs analysis

#### DNA isolation from blood

For DNA isolation from blood samples, the paramagnetic beads based instrument Chemagic MSMI (PerkinElmer Inc., MA, USA) was used. Briefly, Chemagic DNA Blood Kit special (PerkinElmer Inc., Cat.# CMG-703-1, MA, USA) was used for 3 ml of blood following manufacturer instructions. The corresponding Tris–HCl elution buffer available in the kits was used.

#### DNA isolation from frozen tissues

For DNA isolation from tissues sections the paramagnetic beads based instrument Chemagic MSMI (PerkinElmer Inc., MA, USA) was used. Chemagic DNA Blood Kit special (Cat. no CMG-703-1, PerkinElmer Inc., MA, USA) was used for tissue sections but with Proteinase K for tissue (Cat. no 834, PerkinElmer Inc., MA, USA) and Lysis Buffer 1 for tissue (Cat. no 805, PerkinElmer Inc., MA, USA). Between 10 and 18 20-µm sections for frozen tissues OCT were used (the exact number of sections varied with the area occupied by the tissue after hematoxylin staining). Tris–HCl elution buffer available in the kits was used.

#### DNA isolation from cell lines

Cell pellets were used for DNA isolation (10^6^ cells). QIAamp DNA Mini Kit (Cat. no 51304, Qiagen; MD, USA) was used in a Qiacube robot following manufacturer instructions.

#### DNA isolation from blood spot stored in FTA cards

The 1.2 mm Harris Uni-core punch (Whatman, MO, USA) was used to obtain a FTA 1.2 mm disc that was introduced in a 0.2 ml PCR tube. DNA was purified from blood sample contained in the disc using the Whatman^®^ FTA^®^ purification reagent (Cat. no A719978-1EA, Sigma Aldrich, MO, USA) following manufacturer instructions. Briefly, three washes were performed with Whatman^®^ FTA^®^ purification reagent followed by three washes with TE buffer. After TE buffer elimination, the FTA disc was dried during 1 h at room temperature. Dry FTA disc was used directly for multiplex PCR.

### Multiplex PCR for 5 STRs loci detection

Multiplex PCR for 5 STRs loci detection (DXS7132, GATA31E08, DYS390, GATA71D03 and DXS6789) was performed with primer sequences described by other author(Gastier et al. 1995; Sheffield et al. 1995). 50 ng of DNA isolated from cell lines, blood or tissue, or a pre-treated 1.2 mm FTA disc, were amplified using the Type-it Microsatellite PCR kit (Cat. No. 206241, Qiagen; MD, USA) according to the manufacturer instructions. The PCR program used was 1 cycle 95 °C 5 s, 32 cycles (95 °C 30 s, 57 °C 30 s y 60 °C 30 s), 1 cycle 60 °C 30 min, on an Eppendorf AG thermocycler (Eppendorf, Hamburg, Germany). PCR products were analyzed by electrophoresis on a 3% and 25 cm long agarose gel stained with GelRed (Cat. no 41003, Biotium, CA, USA). The GeneRulerTM 100 bp Plus DNA Ladder (Cat nº SM0321, Thermo Scientific, MO, USA) was also run out as size reference and results were visualized on a Chemidoc instrument (Bio-Rad, CA, USA).

### Multiplex PCR for 10 STRs loci detection

Multiplex PCR for 10 STRs loci detection (TH01, TPOX, vWA, Amelogenin, CSF1P0, D16S539, D7S820, D13S317, D5S818, D21S11) was performed using 10 ng of DNA isolated from cell lines, blood or tissue, or a pre-treated 1.2 mm FTA disc, and the GenePrint 10 System (Cat. no B9510, Promega, WI, USA) according to the manufacturer instructions. STRs fragment detection was performed by capillary electrophoresis in a 3130 genetic analyzer (Applied Biosystems, Singapore, Asia) using the POP-7 matrix. Data analysis was carried out with the GemaMapper ID-X (v1.0.1) software (Life Technologies, CA, USA).

## Results

**Fig. 1:**
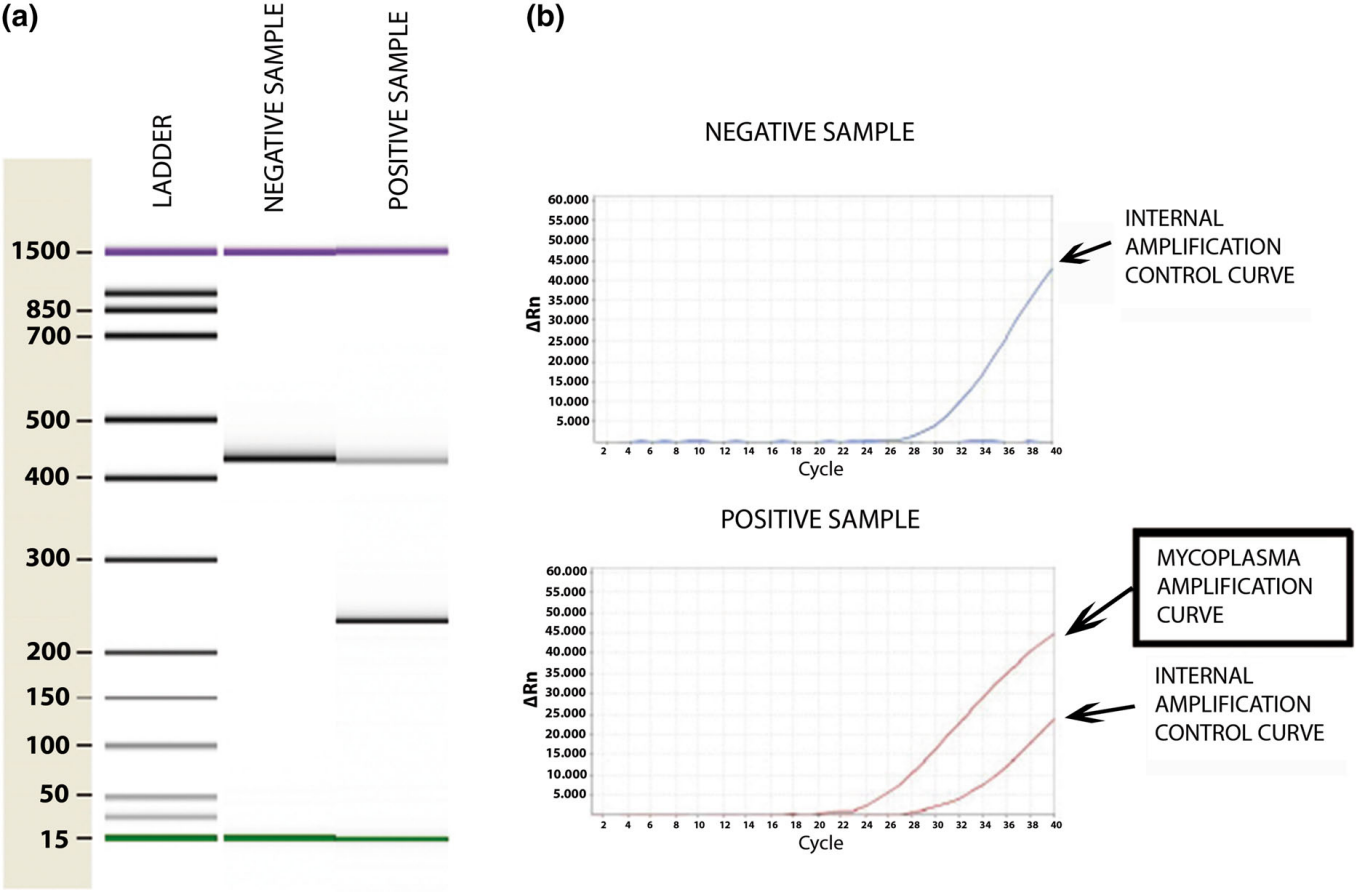
Representative negative and positive results for mycoplasma detection. **a** Conventional PCR + Bioanalyzer, **b** Real-time PCR

Twenty-four cell culture supernatant were used to check and compare both detection method used in the BBSSPA for *mycoplasma* detection by conventional and real time PCR. By conventional PCR a 481 bp band was visualized for internal control detection in negative and positive samples and a specific 260 ± 8 bp band for *mycoplasma* positive samples (Fig. 1a). Specific *mycoplasma* amplification was also observed by real time PCR with internal control detection for all the samples tested (Fig. 1b). With both methods used, five samples from the twenty supernatant samples analyzed were positive for *mycoplasma*. In any case, no invalid results were observed. A *mycoplasma* positive sample visualization by both methods is shown in Fig. 1.

**Fig. 2:**
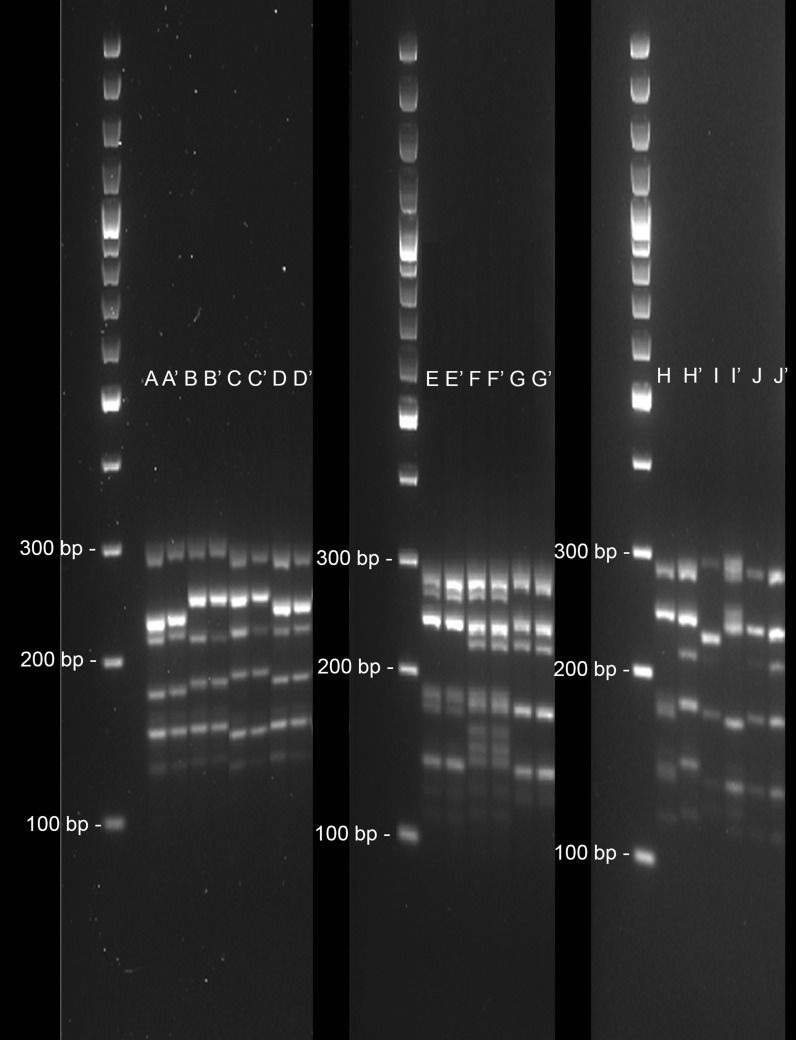
Results obtained using 5 STRs loci detection method. Four cell lines were compared with the original tissue (A and A′, B and B′, C and C′, D and D′), 3 cell lines were compared with frozen blood from the original donor (E and E′, F and F′, G and G′), and 3 cell lines were compared with FTA punch (H and H′, I and I′, J and J′)

STRs analysis by Multiplex PCR for DNA isolated from 10 cell lines, 4 tissues or 3 blood samples, or 3 pre-treated FTA discs, was performed by different methods (5 STRs and 10 STRs loci detection). Corresponding results to the same donor (10 pairs of samples) were compared by both methods: 4 cell lines were compared with the original tissue, 3 cell lines were compared with frozen blood from the original donor, and 3 cell lines were compared with FTA punch. Coincident results were obtained for 5 STRs and 10 STRs loci detection methods except for pre-treated FTA discs, whose results were not precise through 5 STRs loci Multiplex PCR (samples H, I and J, Fig. 2). Clear results were obtained in any case with Multiplex 10 STRs loci detection kit. Results for Multiplex 10 STRs loci detection method is detailed in Table 1.

**Table 1:** Results obtained using multiple 10 Strs detection method

Samples	Analized STRs									
	AMEL	CSF1PO	D13S317	D16S539	D21S11	D5S818	D7S820	TH01	TPOX	VWA
A	X	11, 12	10, 11	8, 9	28, 32.2	11, 13	10, 12	5, 6	9, 12	13, 15
A′	X	11, 13	10, 11	8, 9	28, 32.2	11, 13	10, 12	5, 6	9, 12	13, 15
B	X	10, 12	8, 11	10, 13	29.2, 33.2	11, 12	8, 12	7, 9	8, 11	16, 18
B′	X	10, 12	8, 11	10, 13	29.2, 33.2	11, 12	8, 12	7, 9	8, 11	16, 18
C	X	10, 11	9, 11	8, 10	29, 33.2	11, 12	10, 11	6, 9	8, 11	14, 15
C′	X	10, 12	9, 11	8, 10	29, 33.2	11, 12	10, 11	6, 9	8, 11	14, 15
D	X	11, 12	9, 13	9, 10	29	14, 16	9, 12	6, 10	8, 12	16
D′	X	11, 13	9, 13	9, 10	29	14, 16	9, 12	6, 10	8, 12	16
E	X	12	11, 12	9, 13	31.2, 32.2	10, 12	10, 11	6, 8	8, 10	18
E′	X	12	11, 12	9, 13	31.2, 32.2	10, 12	10, 11	6, 8	8, 10	18
F	X	11, 12	8, 13	10, 11	29, 30	11	9, 11	7, 9	8, 10	18
F′	X	11, 12	8, 13	10, 11	29, 30	11	9, 11	7, 9	8, 10	18
G	X	11, 12	12	11	29, 30	11	9	6, 9	8, 12	15, 18
G′	X	11, 12	12	11	29, 30	11	9	6, 9	8, 12	15, 18
H	X, Y	10, 12	12	12, 13	29, 30	10, 12	10	7	7, 8	16, 18
H′	X, Y	10, 12	12	12, 13	29, 30	10, 12	10	7	7, 8	16, 18
I	X	10, 14	12	9, 11	27, 28	11, 12	8, 9	9	11	15, 17
I′	X	10, 14	12	9, 11	27, 28	11, 12	8, 9	9	11	15, 17
J	X, Y	10	12, 13	8, 14	32.2	9, 12	10, 12	9, 9.3	11	15
J′	X, Y	10	12, 13	8, 14	32.2	9, 12	10, 12	9, 9.3	11	15

Four cell lines were compared with the original tissue (A and A′, B and B′, C and C′, D and D′), 3 cell lines were compared with frozen blood from the original donor (E and E′, F and F′, G and G′), and 3 cell lines were compared with FTA punch (H and H′, I and I′, J and J′)

## Discussion

Contamination by *mycoplasma* and cell lines cross-contamination are recognized as the most serious and persistent problems in mammalian cell lines culture (Geraghty et al. 2014), being a great source of false scientific results (Drexler et al. 2003; Nubling et al. 2015; Drexler et al. 2017). Early cross-contamination of a newly established cell line is usual and can result in the worldwide spread of a misidentified cell line (Chatterjee 2007).

Studies carried out in USA by FDA report that 15% over 20,000 cell cultures were contaminated with *mycoplasma* (Barile 1979). In Europe, *mycoplasma* contamination levels detected were over 25% of 1949 cell cultures from the Netherlands and 37% of 327 cultures from Czechoslovakia (McGarrity 1988). The incidence of *mycoplasma* contamination was reported to be 57.5% in Iran (Molla Kazemiha et al. 2014), 80% in Japan (Koshimizu and Kotani 1981) and 88.7% in Mexico (Rivera et al. 2009). Published data from the German Cell Lines Bank DSMZ inform that 187 from 598 leukemia-lymphoma cell lines (31%) were contaminated with *mycoplasma* (Capes-Davis et al. 2010) and recent studies show a ratio of 24/82 cell cultures contamination (29.3%) (Falagan-Lotsch et al. 2015). These disturbing data are due to absent or inadequate testing in many laboratories (Capes-Davis et al. 2010).

Otherwise, cell line misidentification is one of the most serious and persistent problem in culture labs originates from cross-contamination with another cell line (Geraghty et al. 2014; Huang 2017). Usually cross-contamination may occur at the beginning of the cell line generation, so never has exist the pure cell line, and it’s impossible to have it. This fact is high frequent because cultures can remain in crisis for a prolonged period of time before emerging as an immortalized line; in this period a foreign cell line can be introduced in the culture and proliferate (Capes-Davis et al. 2010).

Misidentification problem dates from 1950s. Between 16 and 35% of cell lines used in experiments have been misidentified or cross-contaminated with other cell lines (Reid et al. 2013). Specifically, 18% of 252 submitted cell lines at German Cell Lines Bank DSMZ were misidentified (Capes-Davis, Theodosopoulos et al. 2010) and 95 of 380 cell lines (25% of cross-contamination) used in China (Ye et al. 2015). Curiously, 93.22% of the foreign cells detected in China were HeLa cells. Recently, 46.0% (128/278) of misidentification for a panel of 278 cell lines from 28 institutes in China has been described by comparing the DNA profiles with the cell bank databases of ATCC and DSMZ (Huang et al. 2017). From 2012 to 2014, a 13.8% of misidentification was detected over 111 cell line authentication test performed by Cell Line Authentication Service at Brazilian Metrology Institute in Brazil, (Cosme et al. 2017). Results derived from misidentified lines have been published in thousands of articles and have been used in drugs screening leading to unusable or even harmful therapeutic strategies (Ye et al. 2015).

In spite of previous results, different works show the high frequency of cell lines distribution between laboratories versus the limited tests performed for *mycoplasma* and authentication. According to a 2004 survey, 63% of researchers (n = 485) have acquired at least one cell line from another laboratory, while 45% have never tested their cell lines for authenticity (Buehring et al. 2004). A 2013 survey disclose that 25% (n = 250) of laboratories do not perform *mycoplasma* test (Shannon et al. 2016). Data from this same survey show that 76% (n = 111) of users obtained cell lines from other laboratories where *mycoplasma* and authentication tests are not frequently performed, and only 46% (115/250) of researchers that typically perform authentication testing in their laboratory (Shannon et al. 2016). Only 39.89% (79/198) and 69.19% (137/198) of researchers perform *mycoplasma* and authentication analysis in samples managed recently respectively, and 74.8% (187/250) and 46% (115/250) of researchers have decided to perform *mycoplasma* and authentication analysis respectively, in the future (Shannon et al. 2016).

The World Health Organization propose to harmonize assays for *mycoplasma* DNA detection (WHO 2014). A large number of methods with different properties of sensitivity and specificity for *mycoplasma* testing are available: microbiological culture, direct DNA staining, biochemical detection and Nucleic Acid Amplification Techniques (NAT assays) (Geraghty et al. 2014). Although microbiological culture has been the “gold standard” for detection of viable *mycoplasma*, the overall testing strategy is time consuming (a minimum of 28 days) (Duke et al. 1966). The most extended and sensitive but not the cheapest methods for *mycoplasma* testing are NAT assays with their different variations: quantitative, semiquantitative or qualitative (Sheppard et al. 2009). NAT assays allow to have results in 2–3 h by using real-time PCR, the specificity is really high, and detect most of the Mollicutes species.

Two different NAT assays were selected for *mycoplasma* testing. Valid and coincident results were obtained with both conventional and real time PCR based methods. But important advantages are listed for real-time PCR comparing conventional PCR. Real-time PCR method is able to detect 66 different *mycoplasma* species whereas conventional PCR method only detects 19 (Table 2). The lower manipulation in real-time PCR assays, linked to the fact that in the real-time PCR method, PCR amplified tubes are never opened in the laboratory, reduces drastically the risk of contamination. Real-time PCR results are semi-quantitative being indicative of the grade of *mycoplasma* contamination in the cell culture. Additionally, real-time PCR method interpretation is easier thanks to the numeric value obtained and results interpretation from Agilent chip by technicians is not necessary. Finally and not less important, analysis prize per sample is lower for real-time PCR method.

**Table 2:** Features For conventional and real-time PCR mycoplasma detection methods

	PCR LookOut Mycoplasma pcr detection kit (Sigma-Aldrich) + Agilent Bionalizer	PCR LookOut Mycoplasma qPCR detection kit (Sigma-Aldrich)
Valid results obtained	100%	100%
Sensitivity	4–40 genome copies per assay	4–40 genome copies per assay
Number of species	19	66
Manipulation	High	Low
Result	Qualitative	Quantitative
Interpretation complexity	Medium	Easy. Numeric result (Ct value)
Cost/assay	23.73 €	19.37 €

Summary of special features for conventional PCR LookOut Mycoplasma PCR detection kit (Sigma Aldrich) and Agilent Bionalizer visualization, compared with real-time LookOut Mycoplasma qPCR detection kit (Sigma Aldrich)

The concept of biochemical polymorphisms was introduced in 1966 to distinguish human cell lines on the basis of their isozymes expression (Gartler 1967). Previously in 1962 the first bank of authenticated cell lines was established at the ATCC using karyotyping and immunological approaches (Christine Alston-Roberts et al. 2010). Currently, the Short Tandem Repeats (STR) profiling is the reference method for cell line identification (Mehta et al. 2017), and standard STRs profiling protocols have been established by ATCC SDO workgroup ASN-002 for cell line authentication (Christine Alston-Roberts et al. 2010). The presence of STRs within the human genome exists at variable lengths throughout the population. A cell line is considered authentic when the STR profile shows at least 80% matching with the original tissue or its derivatives (Rubocki et al. 2000).

Different starting material (blood, tissue and FTA punches) for DNA isolation was used to validate the Multiplex PCR methods through the distinct steps of cell line generation process. When we used 5 STRs and 10 STRs loci detection for checking cell lines authentication comparing to tissue and blood samples from the corresponding donors, good results were obtained of sensitivity and reliability. However reliable results were obtained for FTA punches tested with the 10 STRs Multiplex PCR method but not with the 5 STRs Multiplex PCR method. We hypothesize that it can be due to low DNA concentration in FTA punches, being probably the 10 STRs Multiplex PCR method more robust, sensitive and reliable for this kind of samples because of less DNA quantity required for STRs detection. Although technique complexity is higher, the fingerprint using 10 STRs loci provides an exact, sensitive, precise and objective result through capillary electrophoresis in an analyzer (Table 3), which allows comparing DNA fingerprints across several experimental runs and sharing between laboratories and public online databases (Romano et al. 2009). On the contrary, the 5 STRs PCR method requires training of technician for low resolution agarose gel interpretation and the obtained results will always be subjective. The main disadvantage of 10 STRs Multiplex PCR method versus 5 STRs loci detection method is the higher costs per assay (Table 3) but Multiplex 10 STRs loci detection method has been recognized and approved by American National Standards Institute (ANSI) and American Type Culture Collection (ATCC).

**Table 3:** Advantages And disadvantages for both STRS analysis methods

	5 STRs detection method	10 STRs detection method (Geneprint 10 System, Promega)
Discrimination between individual samples	Yes	Yes
STRs analysed	5	10
Methodology	PCR + Agarose electrophoresis	PCR + Capilar electrophoresis
Tecnich complexity	Medium	High
Interpretation	Sometimes a bit subjective, depending user expertise	Objective (high sensitivity and resolution)
Cost/assay	13.07 €	47.97 €

Advantages and disadvantages summary analysis for both detection methods: 5 STRs method and 10 STRs method (Geneprint 10 System, Promega)

Currently, prestigious scientific journals require evidence of cell lines authentication and absence of cross-contamination before data publication using immortalized cell lines (Lichter et al. 2010) as well as evidence of absence of contamination by *mycoplasma* (Hancocks 2013). However, examples such as the misidentified NCI/ADR-RES cell lines have been revealed, which were used for publishing around 300 papers (Liscovitch and Ravid 2007), or clinical trials and patents described using misidentified cell lines (Boonstra et al. 2010). By other hand, some top peer-reviewed journals present publications having some of the most contaminated series of cells with *mycoplasma* (Olarerin-George and Hogenesch 2015).

In conclusion, *mycoplasma* detection and authentication by validated methods of newly established or received cell lines prior to entering cell line collections is an essential issue. Literature and cell bank websites revision to find information about previous cross-contamination, and periodically testing of cell lines before cryopreservation and when thawed from liquid nitrogen is considered a good cell culture practice (Freshney 2010). In case of biobanks, cell lines’ checking is mandatory to provide a high-quality bioresource for research. Biobanks have to implement a consistent system for guaranteeing the authentication and to avoid the spreading of misidentified cells lines as well as the absence of *mycoplasma* in the supplied cell lines. This is the reason because highly recommended methods have been routinely introduced in the SSPA’s biobank for *mycoplasma* contamination and cell line authentication testing.
